# Supplementary material for: Structural Transformation and Creativity Induced by Biological Agents during Fermentation of Edible Nuts from Terminalia catappa
Source: Molecules. 2021 Sep 28;26(19):5874. doi: 10.3390/molecules26195874 (PMC8510340; doi:10.3390/molecules26195874)
Supplement: Supplementary file 1 [file molecules-26-05874-s001.zip › MS T catappa Supplementary TABLES.pdf]

Table S1. The GC-MS detected phytochemicals in the unfermented aqueous extract of *T. catappa* seeds.

Peaks	RT	Area((%)	Similarity Index(%)	Class of Compound	IUPAC Name	Common Name
1	5.025	8.67	82	Carboxylic acid ester	3-Methyloxirane-2-carboxylic acid	trans-2,3-Epoxy-buttersaure
2	5.733	0.81	73	Fatty acid	(E)-hex-3-enedioic acid	3-Hexenedioic acid
3	5.917	1.24	82	Alcohol	hexane-2,3,4,5-tetrol	1,6-Dideoxy-l-mannitol
4	6.828	0.56	68	Fatty acid	2-amino-4-(2-amino-2-carboxyethyl)sulfanyl-butanoic acid	dl-Allo-cystathionine
5	7.775	8.74	81	Alkylglycerol	3-(2,3-dihydroxypropoxy)propane-1,2-diol	Diglycerol
6	8.082	0.55	72	Fatty acid	4-(4-methoxyphenoxy)-4-oxobutanoic acid	4-Methoxyphenyl Hydrogen Succinate
7	8.917	2.04	84	Pyrrolidinone	pyrrolidin-2-one	2-Pyrrolidinone
8	8.696	1.93	70	Alcohol	1-Methoxy-3-hydroxymethylheptane	2-butyl-4-methoxybutanol
9	9.100	1.30	77	Ketone	3,5-Dihydroxy-6-methyl-2,3-dihydro-4H-pyran-4-one	2,3-Dihydro-3,5-dihydroxy-6-methyl-4h-pyran-4-one
10	10.200	0.47	68	Amino acid	S-[2-Aminoethyl]-dl-cysteine	Thiosine
11	10.368	1.34	74	Carboxylic acid ester	1-(1-Hydroxy-1-methyl-ethyl)-cyclobutanecar-boxylic acid	1-(2-hydroxypropan-2-yl)cyclobutane-1-carboxylic acid
12	10.501	1.97	74	Fatty acid	(2Z)-2-Octenoic acid	2-Octenoic acid
13	10.790	0.47	69	Carboxylic acid ester	1-Di(tert-butyl)silyloxydodecane	Di(tert-butyl)silyl dodecyl ether
14	10.995	0.17	67	Amide	5-O-Methyl-d-gluconic acid dimethylamide	5-O-Methyl-d-gluconic acid dimethylamide
15	11.391	2.85	63	Alkane	Sulfide, cyclohexyl isopentyl	Sulfide, cyclohexyl isopentyl
16	11.827	6.03	89	Phenol	Phenol, 2,6-dimethoxy-	Syringol
17	12.249	0.44	70	Fatty acid	6-amino-6-oxohexanoic acid	Adipamic acid
18	12.318	1.71	90	Amino acid	(2S)-2,5-diamino-5-oxopentanoic acid	Glutamine
19	12.668	0.31	68	Carboxylic acid	(1R,2R)-cyclobutane-1,2-dicarboxylic acid	1,2-Cyclobutanedicarboxylic acid, trans-
20	13.633	1.79	89	Fatty acid	methyl dodecanoate	Methyl laurate
21	14.024	1.42	71	Ketone	6-hydroxy-3,3a,4,5,6,6a-hexahydrocyclo-penta[b]furan-2-one	6-Hydroxyhexahydrocyclopenta[b]furan-2-one
22	14.102	2.43	61	Ketone	3-[(E)-pent-2-enyl]cyclopentane-1,2,4-trione	1,2,4-Cyclopentanetrione, 3-(2-pentenyl)-

23	14.205	3.52	72	Carboxylic acid ester	1-(1-Hydroxy-1-methyl-ethyl)-cyclobutanecarboxylic acid	1-(2-hydroxypropan-2-yl)cyclobutane-1-carboxylic acid
24	14.328	2.62	63	Carboxylic acid ester	3,3-dimethyl-4-oxocyclopentane-1-carboxylic acid	3,3-Dimethyl-4-oxocyclopentanecarboxylic acid
25	14.535	0.95	70	Fatty acid	6-amino-6-oxohexanoic acid	Adipamic acid
26	14.634	1.23	70	Amine	N-[6-(aziridin-1-yl)hexyl]cyclooctanamine	N-[[6-Cyclooctylaminohexyl]aziridine
27	14.686	1.72	62	Carboxylic acid ester	[(6E,8E)-trideca-6,8-dien-2-yl] acetate	E,E-6,8-Tridecadien-2-ol, acetate
28	14.818	0.93	69	Alcohol	Cyclooctane-1,4-diol, cis	1,4-Cyclooctanediol
29	14.980	0.73	64	Fatty acid ester	methyl 8-[2-(2-hexylcyclopropyl)cyclopropyl] Octanoate	[1,1'-Bicyclopropyl]-2-octanoic acid, 2'-hexyl-, methyl ester
30	15.389	1.61	83	Fatty acid ester	methyl 12-methyltridecanoate	Methyl isomyristate
31	15.593	1.79	70	Amine	N-[6-(aziridin-1-yl)hexyl]cyclooctanamine	N-[[6-Cyclooctylaminohexyl]aziridine
32	15.728	2.74	67	Fatty acid	(E)-undec-2-enoic acid	2-Undecenoic acid
33	15.826	1.93	71	Ketone	4,4,7a-trimethyl-3a,5,6,7-tetrahydro-3H-1-benzofuran-2-one	Tetrahydroactinidiolide
34	16.334	1.86	60	Ketone	2-Methoxy-4,4-dimethyl-2-cyclohexen-1-one	2-methoxy-4,4-dimethylcyclohex-2-en-1-one
35	16.631	0.89	65	Fatty acid	(E)-undec-2-enoic acid	Trans-2-Undecenoic acid
36	16.870	1.53	90	Fatty acid ester	methyl hexadecanoate	Methyl palmitate
37	17.045	2.19	80	Fatty acid	(Z)-octadec-9-enoic acid	oleic acid
38	17.165	5.20	94	Fatty acid	hexadecanoic acid	palmitic acid
39	17.744	2.80	85	Fatty acid	cis-9-Hexadecenoic acid	palmitoleic acid
40	18.034	2.97	91	Fatty acid ester	methyl (Z)-octadec-9-enoate	Methyl oleate
41	18.179	2.36	68	Fatty acid ester	methyl undec-10-ynoate	Methyl 10-undecynoate
42	18.314	3.57	84	Aldehyde	tetradec-13-enal	13-Tetradecenal
43	18.433	0.94	75	Fatty acid	methyl 8-[2-(2-hexylcyclopropyl)cyclopropyl]octanoate	[1,1'-Bicyclopropyl]-2-octanoic acid, 2'-hexyl-, methyl ester
44	18.577	0.46	75	Fatty acid ester	Cyclopropaneoctanoic acid, 2-[[2-[(2-ethylcyclopropyl)methyl] cyclopropyl]methyl]-, methyl ester	Methyl 8-[2-((2-[(2-ethylcyclopropyl)methyl]cyclopropyl)methyl) cyclopropyl]octanoate
45	18.990	0.51	70	Fatty acid	17-Octadecynoic acid	Alkynyl Stearic Acid

46	19.117	2.99	74	Amine	N-[2-(aziridin-1-yl)ethyl]cyclooctanamine	N-[2-[Cyclooctylamino]ethyl]aziridine
47	19.241	1.19	80	Fatty acid ester	3-hydroxybutan-2-yl octadecanoate	2-Hydroxy-1-methylpropyl stearate
48	19.645	0.44	69	Fatty acid	7-oxooctanoic acid	7-Keto-N-Caprylic Acid
49	19.762	1.57	69	Amine	(Z)-octadec-9-en-1-amine	Oleylamine
50	20.272	0.48	69	Fatty acid ester	9,12,15-Octadecatrienoic acid, 2-[(trimethylsilyl)oxy]-1-[[(trimethylsilyl)oxy]methyl]ethyl ester, (Z,Z,Z)-	2-Monolinolenin, 2TMS derivative
51	20.357	0.64	65	Fatty acid ester	ethyl (Z)-11-(3-ethenylcyclopentyl) undec-10-enoate	11-(3-Ethenylcyclopentyl)undec-10-enoic acid, ethyl ester
52	21.025	0.38	75	Fatty acid ester	9,12,15-Octadecatrienoic acid, 2-[(trimethylsilyl)oxy]-1-[[(trimethylsilyl)oxy]methyl]ethyl ester, (Z,Z,Z)-	2-Monolinolenin, 2TMS derivative

Table S2. The GC-MS detected phytochemicals in the aqueous extract of fermented *T. catappa* seeds.

Peaks	RT	Area (%)	Similarity Index (%)	Class of Compound Class	IUPAC name	Common name
1	4.513	1.81	91	Alcohol	Butane-2,3-diol	2,3-butanediol
2	4.700	2.21	81	Carboxylic acid ester	Acetic acid, ethoxyhydroxy-, ethyl ester	Ethyl glyoxylate hemiacetal
3	4.886	16.20	87	Fatty acid	Butanoic acid	Butyric acid
4	5.292	19.66	89	Alcohol	propane-1,3-diol	Trimethylene glycol
5	6.365	5.42	84	Alcohol	hexane-2,3,4,5-tetrol	1,6-Dideoxy-l-mannitol
6	6.527	3.24	83	Fatty acid	Hexanoic acid	Caproic acid
7	8.348	2.89	85	Alcohol	2,2-dimethylpentan-1-ol	Neoheptanol
8	8.583	1.76	68	Fatty acid ester	11-cyclopent-2-en-1-yl undecanoic acid	Hydnocarpic acid
9	8.912	0.86	82	Fatty acid ester	propyl butanoate	Propyl butyrate
10	9.348	0.31	77	Fatty acid	(Z)-oct-2-enoic acid	2-Octenoic acid
11	9.612	0.19	81	Amide	2,3,4,6-tetrahydroxy-5-methoxy-N,N-dimethylhexanamide	5-O-Methyl-d-gluconic acid dimethylamide
12	10.055	6.63	96	piperidones (delta-lactam)	2-Piperidinone	Valerolactam
13	10.125	0.19	82	Alcohol	1-(4a,6,7,8a-tetrahydro-4H-[1,3]dioxino[4,5-b][1,4]dioxin-4-yl)ethanol	1,3:2,4-Dimethylene-d-epirhamnitol
14	10.342	0.54	65	Fatty acid ester	11-cyclopent-2-en-1-ylundecanoic acid	Hydnocarpic acid
15	10.443	0.24	81	Carboxylic acid ester	4-hydroxy-2-methylpyrrolidine-2-carboxylic acid	4-Hydroxy-2-methylpyrrolidine-2-carboxylic acid
16	10.528	0.78	78	Azine	2H-1,3-Benzoxazine, 6-chloro-3-cyclohexyl-3,4-dihydro-	
17	10.970	0.65	86	Catechol	1,2-Benzenediol, 3-methoxy-	3-methoxycatechol
18	11.069	0.31	72	Carboxylic acid ester	methyl 2,6-dimethyl-4,4a,8,8a-tetrahydro-[1,3]dioxino[5,4-d][1,3]dioxine-4-carboxylate	2,4:3,5-Diethylidene-xylosecarboxylic acid, methyl(ester)
19	11.137	0.36	73	Fatty acid ester	Hexanoic acid, propyl ester	Propyl caproate
20	11.254	0.54	69	Alcohol	6-(hydroxymethyl)oxane-2,3,5-triol	3-Deoxyglucose
21	11.416	1.56	73	Amino acid	2,6-diamino-4-chlorohexanoic acid	4-Chlorolysine
22	11.679	1.38	72	Alcohol	4-(hydroxymethyl)-7-methyl-1,3-dioxepane-5,6-diol	2,5-Methylene-d,l-rhamnitol
23	11.853	2.11	59	Phenol	2,6-dimethoxyphenol	Syringol
24	12.269	1.97	70	Ketone	1-(2-hydroxyethyl)pyrrolidine-2,5-dione	N-(2-Hydroxyethyl)succinimide
25	12.406	1.20	72	Nitrile	bicyclo[4.2.0]octa-2,4-diene-7-carbonitrile	Bicyclo[4.2.0]octa-2,4-diene-7-carbonitrile

26	13.066	0.96	82	Phenol	Phenol, 4-(2-aminoethyl)-	4-(2-aminoethyl)phenol
27	13.633	0.66	86	Fatty acid ester	Dodecanoic acid, methyl ester	Methyl laurate
28	14.111	0.35	66	Silane	trimethyl-(2-methylphenoxy)silane	Silane, trimethyl(o-tolyloxy)
29	14.524	0.51	68	Amide	6-amino-6-oxohexanoic acid	Adipamic acid
30	14.958	0.38	65	Fatty acid ester	Cyclohexyl 3-phenylpropanoate	Cyclohexyl-.beta.-phenylpropionate
31	15.390	0.73	68	Fatty acid ester	Benzenepropanoic acid, heptyl ester	Heptyl 3-phenylpropanoate
32	15.984	2.25	81	Aldehyde	2,3,4,5,6,7-hexahydroxyheptanal	d-Glycero-d-tallo-heptose
33	16.869	0.44	90	Fatty acid ester	Hexadecanoic acid, methyl ester	Methyl palmitate
34	17.082	1.18	91	Fatty acid	cis-9-Hexadecenoic acid	Palmitoleic acid
35	17.191	4.19	94	Fatty acid	hexadecanoic acid	palmitic acid
36	17.542	0.76	71	Ketone	9,9-dimethoxybicyclo[3.3.1]nonane-2,4-dione	9,9-dimethoxybicyclo[3.3.1]nonane-2,4-dione
37	17.761	1.48	87	Fatty acid	cis-9-Hexadecenoic acid	palmitoleic acid
38	18.034	1.12	90	Fatty acid ester	9-Octadecenoic acid (Z)-, methyl ester	Methyl oleate
39	18.171	2.80	86	Amide	Tyramine, N-formyl-	Formamide, N-(p-hydroxyphenethyl)-
40	18.349	1.69	89	Fatty acid	(Z)-octadec-11-enoic acid	cis-Vaccenic acid
41	18.450	0.61	78	Ketone	9,9-dimethoxybicyclo[3.3.1]nonane-2,4-dione	9,9-Dimethoxybicyclo[3.3.1]nona-2,4-dione
42	18.583	0.31	76	Amide	(E)-8-methylnon-6-enamide	8-Methyl-6-nonenamide
43	19.131	7.52	76	Ketone	Piperidine, 1-(4-piperidinylcarbonyl)-	1-(piperidin-4-ylcarbonyl)piperidine
44	19.238	0.58	72	Fatty acid ester	3-hydroxybutan-2-yl octadecanoate	2-Hydroxy-1-methylpropyl stearate
45	19.368	0.17	73	Fatty acid ester	Cyclopropanebutanoic acid, 2-[[2-[[2-[(2-pentylcyclopropyl)methyl]cyclopropyl]methyl]cyclopropyl] methyl]-, methyl ester	Methyl 4-(2-([2-((2-[(2-pentylcyclopropyl)methyl]cyclopropyl)methyl)cyclopropyl]methyl)cyclopropyl) Butanoate
46	19.482	0.59	78	Aldehyde	(Z)-octadec-9-enal	Olealdehyde
47	19.575	0.71	82	Fatty acid ester	Octadecanoic acid, 3-hydroxypropyl ester	Stearic acid, 3-hydroxypropyl ester
48	19.762	2.46	74	Ketone	5-(4-hydroxybutyl)imidazolidine-2,4-dione	Hydantoin, 5-(4-hydroxybutyl)-
49	20.122	0.42	78	Aldehyde	(Z)-hexadec-7-enal	(Z)-7-Hexadecenal
50	20.270	0.57	59	Carboxylic acid ester	(4-methoxy-1,4,4a,5,8,8a-hexahydronaphthalen-1-yl) acetate	Acetic acid,

						4-methoxy-1,4,4a,5,8,8a-hexahydronaphthalen-1-yl ester
51	20.321	0.47	73	Fatty acid ester	methyl 8-[2-(2-hexylcyclopropyl)cyclopropyl]octanoate	[1,1'-Bicyclopropyl]-2-octanoic acid, 2'-hexyl-, methyl ester
52	20.659	0.22	71	Fatty acid	octadec-17-ynoic acid	Alkynyl Stearic Acid
53	20.706	0.43	76	Fatty acid ester	Oleic acid, 3-hydroxypropyl ester	hydroxypropyl oleate
