# Supplementary figures and images for: Structural Transformation and Creativity Induced by Biological Agents during Fermentation of Edible Nuts from Terminalia catappa
Source: Molecules. 2021 Sep 28;26(19):5874. doi: 10.3390/molecules26195874 (PMC8510340; doi:10.3390/molecules26195874)

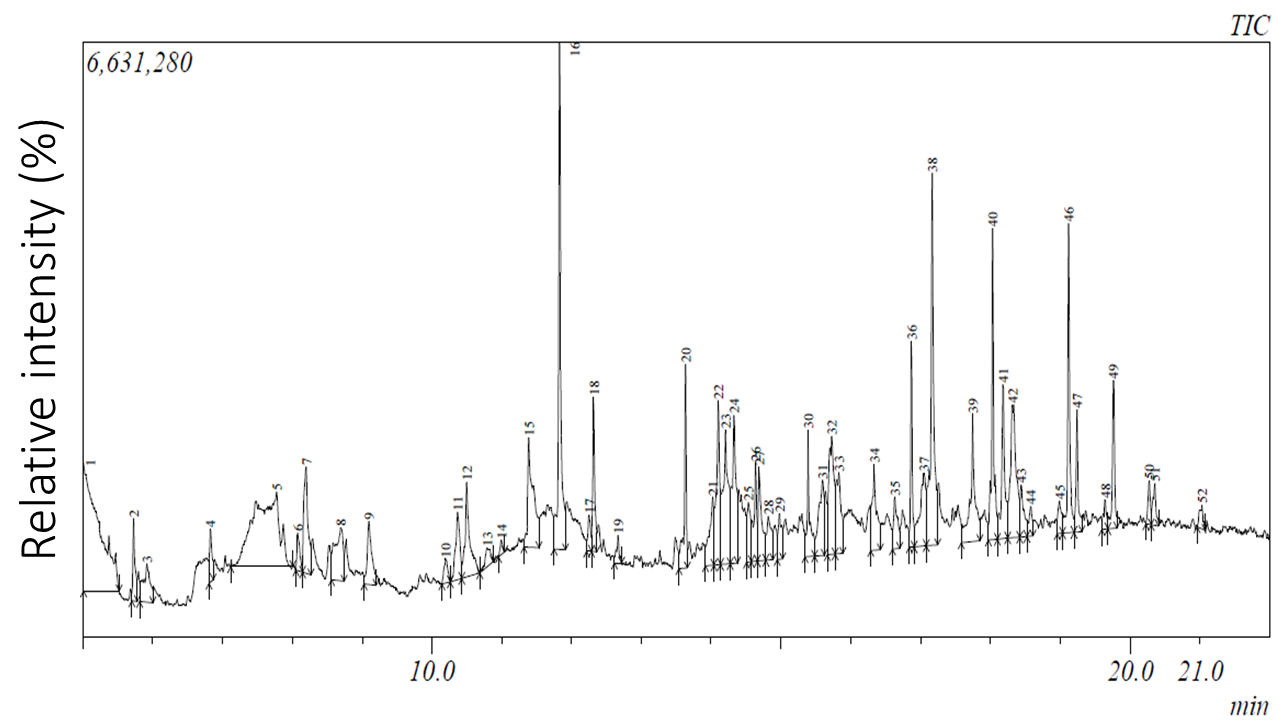

Supplement: Supplementary file 1 [file molecules-26-05874-s001.zip › Figure S1 Unfermented aqueous extract of T Catappa.TIF]

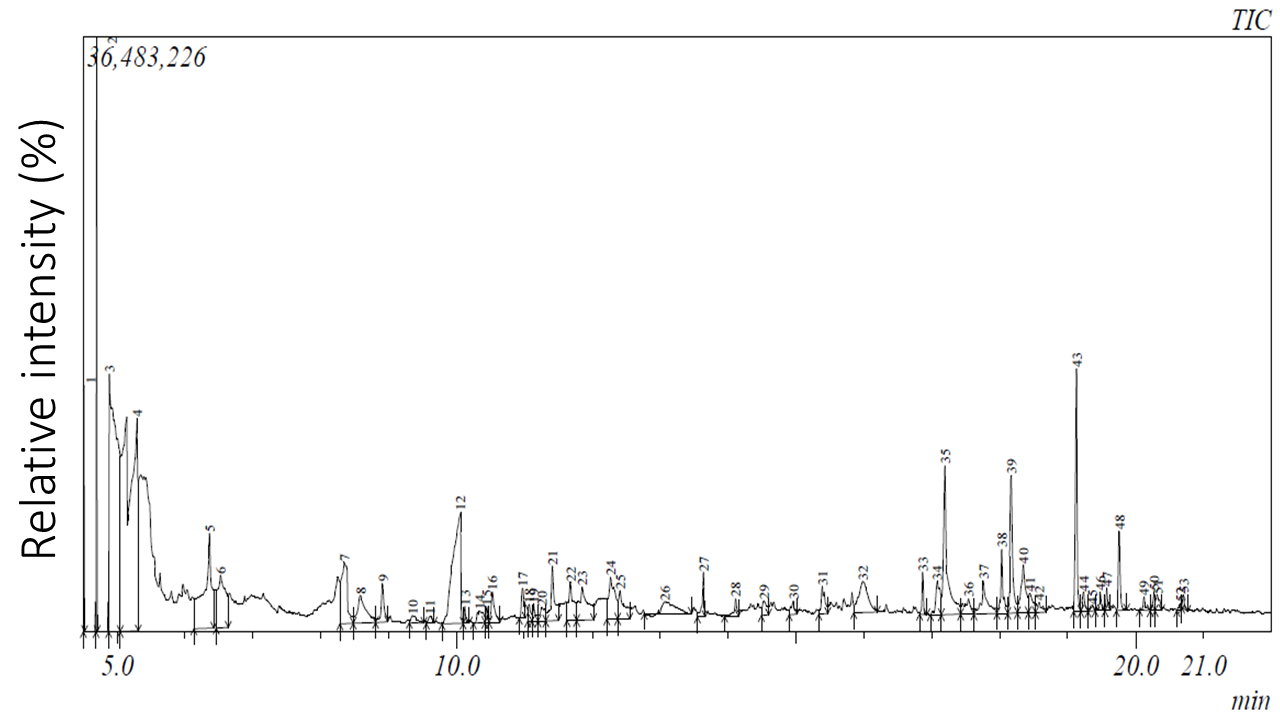

Supplement: Supplementary file 1 [file molecules-26-05874-s001.zip › Figure S2 Fermented aqueous extract of T Catappa.TIF]
